# Adaptive Cognitive Mechanisms to Maintain Calibrated Trust and Reliance in Automation

**DOI:** 10.3389/frobt.2021.652776

**Published:** 2021-05-24

**Authors:** Christian Lebiere, Leslie M. Blaha, Corey K. Fallon, Brett Jefferson

**Affiliations:** ^1^ Department of Psychology, Carnegie Mellon University, Pittsburgh, PA, United States; ^2^ 711th Human Performance Wing, Air Force Research Laboratory, Pittsburgh, PA, United States; ^3^ Pacific Northwest National Laboratory, Richland, WA, United States

**Keywords:** cognitive architectures, ACT-R, trust in automation, automation transparency, trust calibration, human–machine teaming

## Abstract

Trust calibration for a human–machine team is the process by which a human adjusts their expectations of the automation’s reliability and trustworthiness; adaptive support for trust calibration is needed to engender appropriate reliance on automation. Herein, we leverage an instance-based learning ACT-R cognitive model of decisions to obtain and rely on an automated assistant for visual search in a UAV interface. This cognitive model matches well with the human predictive power statistics measuring reliance decisions; we obtain from the model an internal estimate of automation reliability that mirrors human subjective ratings. The model is able to predict the effect of various potential disruptions, such as environmental changes or particular classes of adversarial intrusions on human trust in automation. Finally, we consider the use of model predictions to improve automation transparency that account for human cognitive biases in order to optimize the bidirectional interaction between human and machine through supporting trust calibration. The implications of our findings for the design of reliable and trustworthy automation are discussed.

## 1 Introduction

The interpersonal trust literature asserts that trust in another person is influenced by indicators of trustworthiness such as loyalty (John et al., 1984), integrity (Mayer et al., 1995), and competence (Grover et al., 2014). Similarly, machines can be seen as having trustworthiness characteristics such as capability, robustness, and reliability that influence an operator’s trust in the tool. Ideally, the operator can accurately assess these characteristics and develop well-calibrated trust (Lee and See, 2004).

According to Lee and See (2004), trust calibration is part of a closed-loop process that influences an operator’s intentions and, ultimately, decisions to rely on the automation (or not). In their process model, trust in the automation evolves based on performance feedback from the technology, organizational influences, cultural differences, and one’s propensity to trust. Although all of these factors likely influence trust evolution, a recently developed cognitive model of trust calibration suggests system feedback plays a particularly powerful role in trust calibration (Blaha et al., 2020).

Poor trust calibration occurs when there is a gap between the operator’s trust and the actual trustworthiness of the tool (Lee and See, 2004). This gap can either be the result of underestimating the tool’s trustworthiness or overrating the tool’s capability, robustness, or reliability. Regardless of the direction, poor trust calibration presents a vulnerability in the human–machine system. In the risk literature, vulnerability has been defined as a “physical feature or operational attribute that renders an entity, asset, system, network, or geographic area open to exploitation or susceptible to a given hazard” (Risk Steering Committee, 2010, p. 38). In a human–machine system, vulnerabilities can exist within the human, within the machine, or within the interaction between the two. Human vulnerabilities such as stress (Matthews, 2021) may impact the perceptual and cognitive abilities of the operator leaving the human component of the system open to threats. Machine vulnerabilities such as poor automation reliability (Chavaillaz et al., 2016) place strain on the human–machine system that may lead to hazards. The organization of vulnerabilities into human and machine categories is similar to the human and technical/environmental antecedent (i.e., genotype) error categories identified in human reliability analysis techniques like cognitive reliability and error analysis method (CREAM) (Phillips and Sagberg, 2014).

Poor trust calibration is an example of a third class of vulnerability in that it is not strictly associated with the human or the tool. This vulnerability is instead a dysfunctional interaction between the human and machine that can lead to performance breakdown. For example, poor trust calibration can lead to automation bias, if the operator overestimates the tool’s performance and begins substituting the automated aid’s recommendation in place of their own vigilance and careful analysis (Mosier et al., 1996). This bias is triggered when the automated decision aid provides an incorrect recommendation that is accepted by the operator without scrutiny. Wickens et al. (2015) found that automation bias may be more detrimental to performance when compared to other human–machine interaction problems such as operator’s complacency.

Underestimating the tool’s trustworthiness may also lead to a decline in tool use or even a complete breakdown in the human–machine system. Research on operator’s response to alarm systems with high false alarm rates illustrates the negative impact of low trust on the human–machine system. Alarms that generate a high number of false alarms can cause the operator to distrust the technology leading to a reduction in compliance and possibly a complete rejection of the technology (Bliss and Fallon, 2006). For example, Seagull and Sanderson (2001) examined alarm response in an anesthesia environment and found that many of the alarms were perceived as false and ignored by the health care workers. Although in some instances ignoring the alarm is the appropriate response, this pattern of behavior may lead the operator to ignore an actual threat (i.e., true alarm). Lack of trust also appears to reduce a user’s intention to adopt new technology (Kim et al., 2009; El-Masri and Tarhini, 2017).

Many operators recognize the dangers of poor trust calibration and work to properly calibrate their perception of the machine’s trustworthiness indicators to its actual capabilities and limitations. Well-calibrated trust requires that the operator receives accurate information about the tool which can come through a variety of channels such as feedback on tool performance, training, or conversations with other users. One approach is to include this important information as part of the tool’s interface. This approach transforms tools from a “black box” to a more transparent machine. Transparency may include information about the underlying analytical principles of the tool, about when the tool might struggle to perform, and about its intended functionality (Lyons, 2013).

Human factor practitioners advocate for more transparent display design to accelerate proper trust calibration. For example, Wickens et al. (2015) recommend automated decision aids that include transparency about the confidence of their recommendations as a way to reduce automation bias. Unfortunately, the interfaces that communicate critical transparency information present an opportunity for adversaries to disrupt the trust calibration process and exploit this vulnerability.

Adversaries can attack the human–machine system in a variety of ways to induce poor trust calibration. One attack is to simply remove one or more sources of transparency the operator relies on for calibrating trust. Another technique is to create a dissociation between the tool’s actual performance and what the system is communicating about its performance. One way the adversary might create this dissociation is by disrupting the tool’s performance, such as the accuracy of its recommendations, without triggering the associated change in transparency displayed on the interface. When employing this attack, the adversary is likely unable to disrupt system performance continuously or permanently. Instead, the adversary may be limited to a few attacks executed strategically. An adversary may decide to attack the machine to create this dissociation in a concentrated series of consecutive attacks in an attempt to create a forceful blow to trust calibration. Another approach might be to attack intermittently over a longer period of time in an attempt to slowly create doubt in the operator. Cognitive modeling work into attacker–defender behavior has investigated various attacks and defense maneuvers (Cranford E. et al., 2020). Similar modeling approaches may be used to model the impact of various attack strategies on operator trust calibration.

We can model a human’s response to various transparency cues to simulate how humans would respond to such attacks. Modeling will allow us to investigate which conditions might cause the greatest disruption to trust calibration. Modeling is a low-cost, fast-turnaround approach for exploring various research questions. The findings from our various models can inform the development of specific hypotheses that can be tested with future human subjects’ research.

We are interested in exploring the following research questions by modeling human interaction with an unreliable automated aid.

1. *How does the removal (or addition) of transparency information from an automated assistant impact trust calibration?* The adversary may disrupt trust calibration by removing transparency information provided to the operator. Without impacting underlying system performance, simply removing transparency cues can create a less transparent system that leads to inappropriate trust and reliance. We can model the short-term and long-term effects of removing transparency cues on human reliance and trust.
2. *Some attacks may be designed to create a disassociation between machine performance and transparency information. How does the scope and timing of these attacks impact trust calibration and performance?* The adversary may have limited resources and is, therefore, unlikely to disrupt machine performance continuously or permanently. One goal of the adversary may be to adjust the timing of attacks to maximize their impact on reliance and trust. For example, several consecutive attacks to a system’s performance may impact trust differently than the same number of attacks distributed randomly over a long period of operation. Modeling human reliance and trust to various attack distributions may provide insights into how the attack timing impacts trust and reliance.

## 2 Materials and Methods

### 2.1 Model of Reliance Calibration

We adopt the perspective that an operator’s calibration of trust and reliance is the result of learning and memory for experiences with an automation technology or system, that is, calibration does not require a specialized cognitive mechanism. Rather, the sum of an observer’s experiences over time with a system serve to shape their expectations about the system’s capabilities and likely performance in different situations. This is consistent with the process description in Lee and See (2004). Computational cognitive architectures simulate human mechanisms of learning and memory, under realistic constraints of human information processing. We can use these computational models to simulate operator decision-making about use and reliance on automation or other system features under different task demands or changing task constraints over time.

**FIGURE 1:**
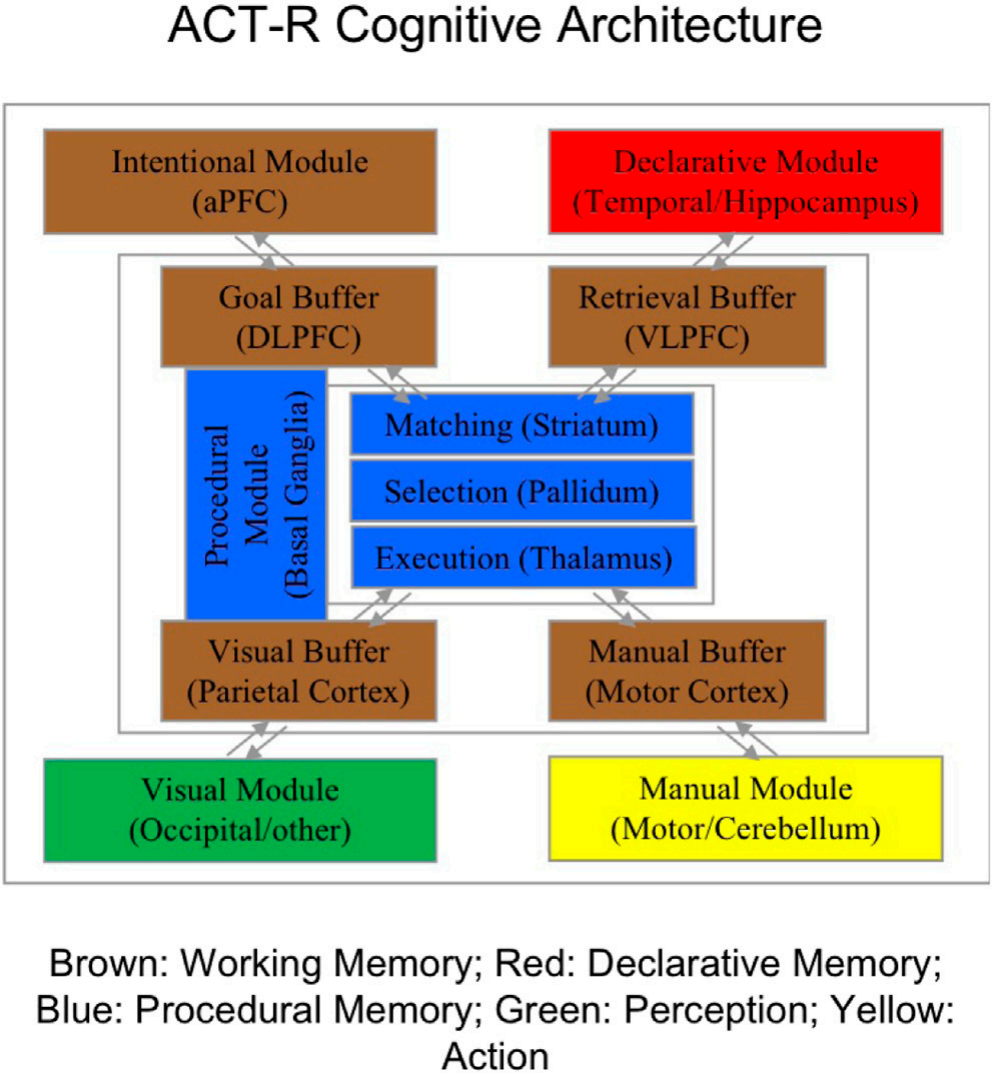
Diagram of the adaptive control of thought-rational (ACT-R) computational cognitive architecture. Each module indicates the neural areas associated with the cognitive mechanisms. The general functionality of the modules is indicated by the color coding.

In Blaha et al. (2020), we developed a computational model of the trust and reliance calibration process using instance-based learning theory (Gonzalez et al., 2003; Gonzalez and Dutt, 2011) integrated into the ACT-R computational cognitive architecture (Anderson et al., 2004). Figure 1 provides a diagram of the various cognitive modules and mechanisms available in the ACT-R architecture. Various modules formally represent information in perception and memory stores, together with the operator’s goals and intentions in the task stored in working memory. The central executive functions provide production rules for the ways in which perceptual and memory inputs interact with goals and intentions to make decisions and execute actions in the environment.

Instance-based learning theory (IBLT), combined with the ACT-R architecture, provides a formal approach for new information to be contextualized by leveraging similar prior experiences and added to memory for use in future decision-making, that is, when someone encounters a new situation for the first time, IBLT provides mechanisms for them to both decide how to react to the present situation and to also integrate the new information into their memory and knowledge to inform future decisions.

We have asserted that calibration results through experiential learning. Experiences are comprised of a person’s observations of cues, the decisions and actions they take, and the feedback they receive about outcomes of those decisions and actions. These prior experiences are stored in memory as a set of instances; instances are structured as the triplet of information {situation cues, choice made, outcome}. According to IBLT, when a person encounters an instance of some situation needing a decision or action (which corresponds to a distinct occurrence of some experience), the person will recall their similar, prior experiences and use those to set expectations about potential outcomes for the current instances. This can be thought of as a process of assessing instance similarity *via* the formal triplet store. IBLT posits that people will make decisions based on the choice that is likely to have the desired or best possible outcome, based on this comparison to prior experiences.

Over time, experiences stored in memory may increase or be strengthened by repeated experiences, while also decaying over time without subsequent exposure. Thus, decisions based on similarity of current instances to those in memory are a function not just of the specific tuples but also the recency and frequency with which a person has had similar experiences or made similar decisions. In the case of automation reliance calibration, this means that if a person has interacted with a system frequently and recently, then representations of the system’s behavior and the operator’s interactions will be readily available and likely heavily weighted in present decisions. In this way, both recent successes and failures of automation can then carry a strong influence on decisions; the salience of past successes and failures will be a function of repeated successes or failures (i.e., similar experiences) building up in memory. And it is through this process of building experience and the salience of desired or undesired outcomes in various situations that reliance calibration emerges through experience.

In our IBLT-based model, we derive an estimate of the reliability of automation (and the alternative options) from the internal state of the model, that is, the internal representation provides the basis for considering the cues and potential choices and setting expectations for the outcome given the features of a specific situation. This expectation is akin to an operator’s calibrated expectation of whether automation will be reliable or perform as intended, given the current circumstances. If this expectation is high enough, in particular higher than alternatives means, the operator will choose to rely on the automation. Otherwise the operator will resort to alternatives such as manual action or the help of human teammates. The outcomes of each decision are then integrated back into memory and informs the computation of future expectations; that is, the calibration process evolves every time a decision is made and is constantly adaptive to the outcome of choices made.

We now describe the formal mechanisms of the IBLT model. Recall that an instance *i* is defined by the triplet {situation cues, choice made, outcome}. Memory instance availability is controlled by activation, $A_{i}$:

$$A_{i}=\text{log}\left({\sum^{\text{​}}}_{j=1}^{n}t_{j}^{-d}\right)+N\left(\sigma \right)$$ (1)

Here, *i* is the memory instance, *d* is the decay parameter controlling the power law of recency, and σ is the magnitude parameter controlling stochastic noise; the summation over all references to that memory provides the power law of practice. Given a particular situation, relevant memories are retrieved by computing their match score that combines their activation with their degree of relevance:

$$M_{i}=A_{i}+{\sum^{\text{​}}}_{j=1}^{l}MP\times Sim\left(d_{j},v_{ij}\right)$$. (2)

Here, *j* is a feature in the situation representation, $d_{j}$ is the corresponding value in the current situation, $v_{ij}$ is the corresponding value in memory *i*, and Sim is the similarity between those two values. Rather than retrieving a single memory, a consensus outcome is generated using the memory blending mechanism satisfying this constraint:

$$V=\underset{V_{j}}{\text{argmin}}{\sum^{\text{​}}}_{i=1}^{k}P_{i}\times Sim{\left(V_{j},v_{ij}\right)}^{2}$$. (3)

Here, *V* is the consensus value among the set $V_{j}$ of possible values and $P_{i}$ is the probability weight of memory *i*, reflecting its match score $M_{i}$ through a Boltzmann softmax distribution.

This consensus value, derived from a weighted blending of prior experiences, sets the expectations for potential outcomes of selected decisions, and guides a decision maker to the option that will be most likely to produce a desired outcome. *V* is not an overt behavior of the system; it is a component of the internal representation in the model of the situation that guides action selection. Blaha et al. (2020) found that querying this blended value after a series of decisions about reliance on automation (i.e., a set of experiences), the model reported a general expectation for decisions about using the automation comparable to the subjective ratings that people had given about the automation system’s reliability after the same amount of experience. *V* computed after some period of time as a blended retrieval with no features specified (i.e., a general, hypothetical rather than specific situation) provided a direct estimate of the calibration of automation reliance. When this calibration metric is comparable to the ground truth of automation reliability, we might generally claim that the model reflects good calibration. When queried under specific situational constraints, the value can indicate if the calibrated model predicts the decision that would be considered accurate (using automation when it is reliable).

### 2.2 Vulnerability in the Model

Model reliance calibration is a function of the experiences with the human–machine system. The situation cues informing the instance representations include both the external signals about the task and any transparency cues that have been designed into the system’s user interface. Likewise, the ability of the human to complete the task, the positive and negative outcomes of their decisions, and the behaviors of the automation can all factor into the instance representation of the outcome of decisions. Here, note that we only represent decisions about the use of automation within the model; decisions about other task aspects, like when to initiate a task or trial, that do not concern use of the automation, do not inform the memory for the automation’s reliability. Because the resulting expectations are a combination of both the transparency cues and the automation’s performance, both dimensions of the system offer opportunities for disruption and miscalibration over experiences. For transparency cues, sources of potential miscalibration are cue values or cue types that are unfamiliar or novel because people may not know how to factor them into the instance representation or might attach to them the wrong interpretation; missing cues that the instance relies upon for judging similarity are another vulnerability. These are directly connected with the instance-based representation of the cues themselves in memory instance *i*. On the behavioral side, after someone has learned which transparency cues should signal particular types of outcomes (e.g., automation acts as desired or automation fails), changes in the model behaviors that deviate from the learned cue-behavior outcomes provide a salient error cue. This will influence the similarity calculations between the current and past experiences, because now there may be transparency cues signaling both positive and negative outcomes that will be factored into *V*. Presence and salience of conflicts over time will influence the calibration process.

We note that *V* can be influenced through these vulnerability mechanisms to produce both over- and under-reliance on automation. If the behavior of the system is signaled to be more reliable than cues indicate, an operator might ignore transparency cues and overuse the automation. Likewise, some negative dissociation between the diagnosticity of cues, or the loss of cues, may garner lower usage of the automation than may be appropriate.

In the following paragraphs, we will explore several ways in which reliance calibration may be influenced through adversarial disruption of a human–machine system. We leverage the IBLT model to explore the degree to which different disruptions influence patterns of decisions to rely on automation and the internal representation of the automation’s reliability through changes of *V* over different conditions.

### 2.3 COBALT Task Environment

**FIGURE 2:**
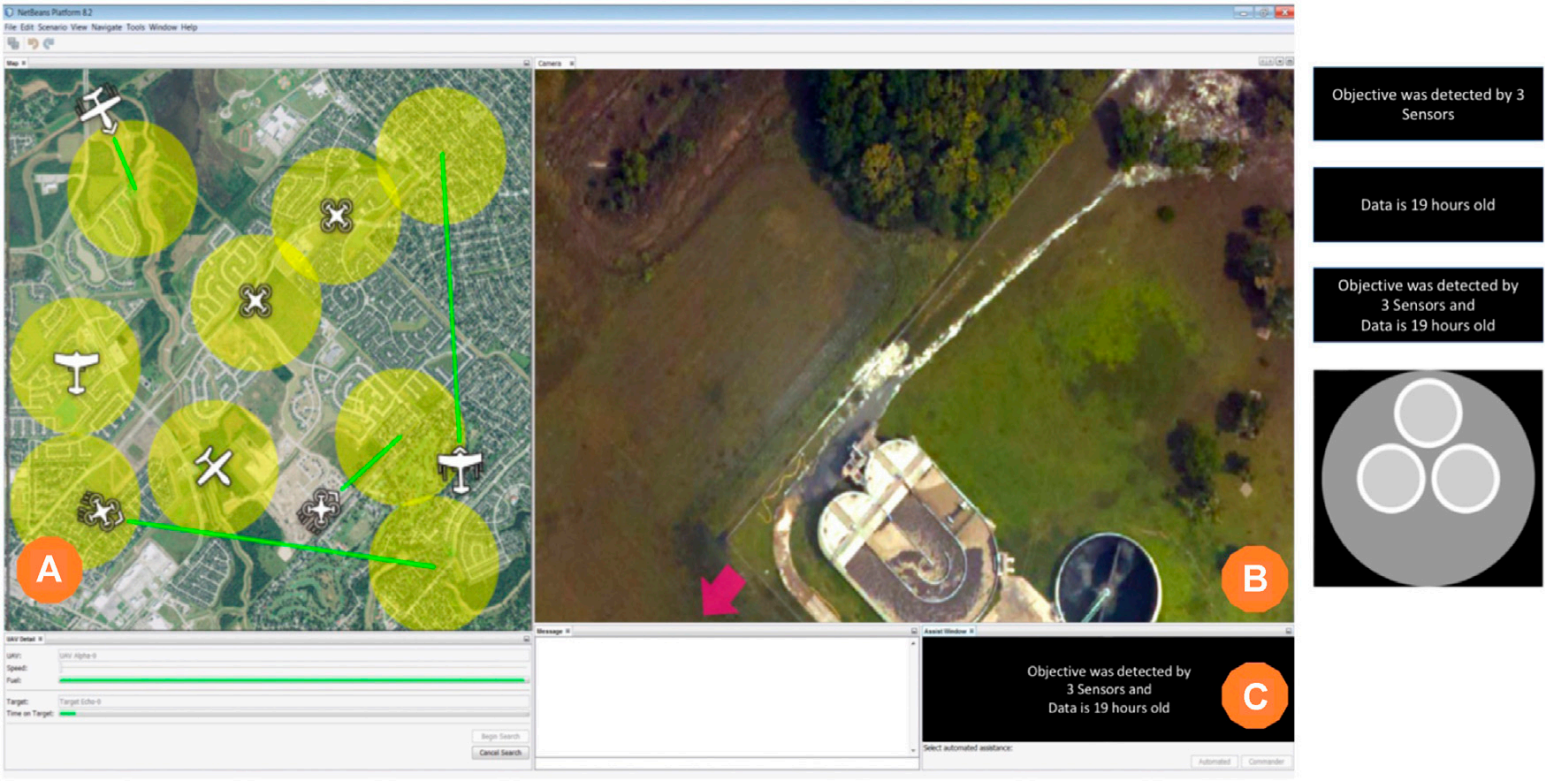
The COBALT task environment. **(A)** marks the UAV map window; users select a UAV to initiate each trial here, **(B)** marks the visual search window, and **(C)** marks the automation transparency cue window. The four images on the right show the four transparency cue types, from top to bottom: number of sensors only, age of data only, age and number in text format (Text condition), and age and number in visual graphic format (Graphic condition).

Our IBL model of automation reliance decisions was developed with empirical data previously collected by this research team in the COBALT task environment (Fallon et al., 2019; Blaha et al., 2020). The COBALT software shown in Figure 2 offers a high-fidelity environment for measuring human decision-making in tasks requiring human–machine teaming, such as monitoring or interacting with automation. The empirical data were taken from an aided visual search task which included two stages on each trial. In the first stage, a user must decide if they would like to have either an Automated assistant or a human Commander assistant provide a search cue to aid their visual search. In the second stage, a user must search through the overhead image (Figure 2B) to find a prespecified target (a red circle) placed on the image.

The search cues were in the form of an arrow on the image potentially pointing in the direction of the target. Cues provided by the Commander were 100% accurate, but there was a 5-s delay before the cue would appear, potentially slowing the search process overall. Participants could wait for the cue, doing nothing, or search unaided while waiting. Cues provided by the Automated assistant were 70% reliable, meaning that in 30% of trials, it would point to some random location in the image other than the location of the target. It was, however, instantaneously available at the start of the trial.

The flow of each trial was as follows. A user initiated a search trial by selecting a UAV in the map window (Figure 2A); note that at the start of a trial, the visual search window (Figure 2B) is “closed,” or shown as a black screen, such that no searching can be completed until a search aid is selected. Each UAV has up to two possible transparency cues that were then given to the user; the purpose of these transparency cues was to provide the user with information that may inform their decision about the reliability of the Automated assistant. The transparency cues were shown in the transparency cue window (Figure 2C); below the transparency cue window are two buttons, Automated and Commander. Users mouse-clicked on the aid of their choice to indicate which search aid type they wanted for that trial. Selection of the search aid instantly initiated the search stage by opening the search window to show the overhead image, appearing like in Figure 2B. Note that although there was a time delay associated with the appearance of the Commander cue, there was no difference in the timing of the start of the search window availability after the assist type selection was made. Participants then searched the visual search window by panning the camera view with the arrow buttons until they located and clicked on the search target.

In the search phase, users can choose to rely on the search cue by panning in the direction the cue indicates; they can choose not to rely on a cue by freely searching in any other direction. If users selected the Commander, they could search unaided or simply wait for the cue; when the Commander cue appeared, users relying on it would follow its direction to reach the target. If users selected and relied on a reliable Automated cue by following it, they could quickly find the target panning in the direction the cue indicated. If users selected and relied on an unreliable Automated cue by following it, the unreliable cue would visibly fail by disappearing from the screen when the participant panned to the false target location. At this time, users could select the Commander button and/or freely search unaided for the target. The longest search times occurred in the task when participants followed an unreliable Automated cue, saw it fail, requested the Commander cue, waited or freely searched unsuccessfully until the Commander cue appeared, and then followed the Commander cue to the target.

### 2.4 Automation Transparency Cues

To help users decide if the Automated assist was going to be reliable, transparency cues were available in the COBALT interface (Figure 2C).

Transparency cues were presented in two formats: text and graphic; examples of all four types are shown to the right of the interface in Figure 2. In the text-based cues, a statement was presented about either the data: age, number of sensors, or both, for example:

**Age:** Data are 15 h old.
**Number:** Objective was detected by two sensors.
**(Age and Number) Text:** Objective was detected by two sensors and data are 15 h old.

We note that this last case is usually referred to as “Text” in our plots and analyses.

The Graphic condition cues always presented both Age and Number data together, like the Text condition. The graphic itself was a circle-packing visualization with the number of circles corresponding to the number of sensors. All circles had a white outline and the brightness of the inner fill color corresponded to the age of the data. A solid white circle was the newest data, 1 h old; the darkest gray fill, matching the background of the circular area, was the oldest data, 36 h old.

The Automated assistant was set to be reliable on 70% of trials. In both Text and Graphic cue conditions, the cues were completely predictive of Automated assist reliability. The Automated assist would be unreliable (point to the wrong location) if age is over 24 h old or the number of sensors is 1. In the Age and Number conditions, the transparency cues appeared more stochastic; they were treated not as a singular cue with deterministic transparency but as multiple cues where one source of information is missing, that is, for the Age condition, there was a small set of trials where data less than 25 h old would result in failed automation because the number cue missing from the display was equal to 1. And for the Number condition, there were a small number of trials where the Automation failed for two or three sensors, because the missing age cue was greater than 24 h old. But across all conditions, the baseline experiment held the reliability rate at 70% reliable and 30% over all trials.

### 2.5 Model Representation of COBALT Automation Reliance Decisions

**FIGURE 3:**
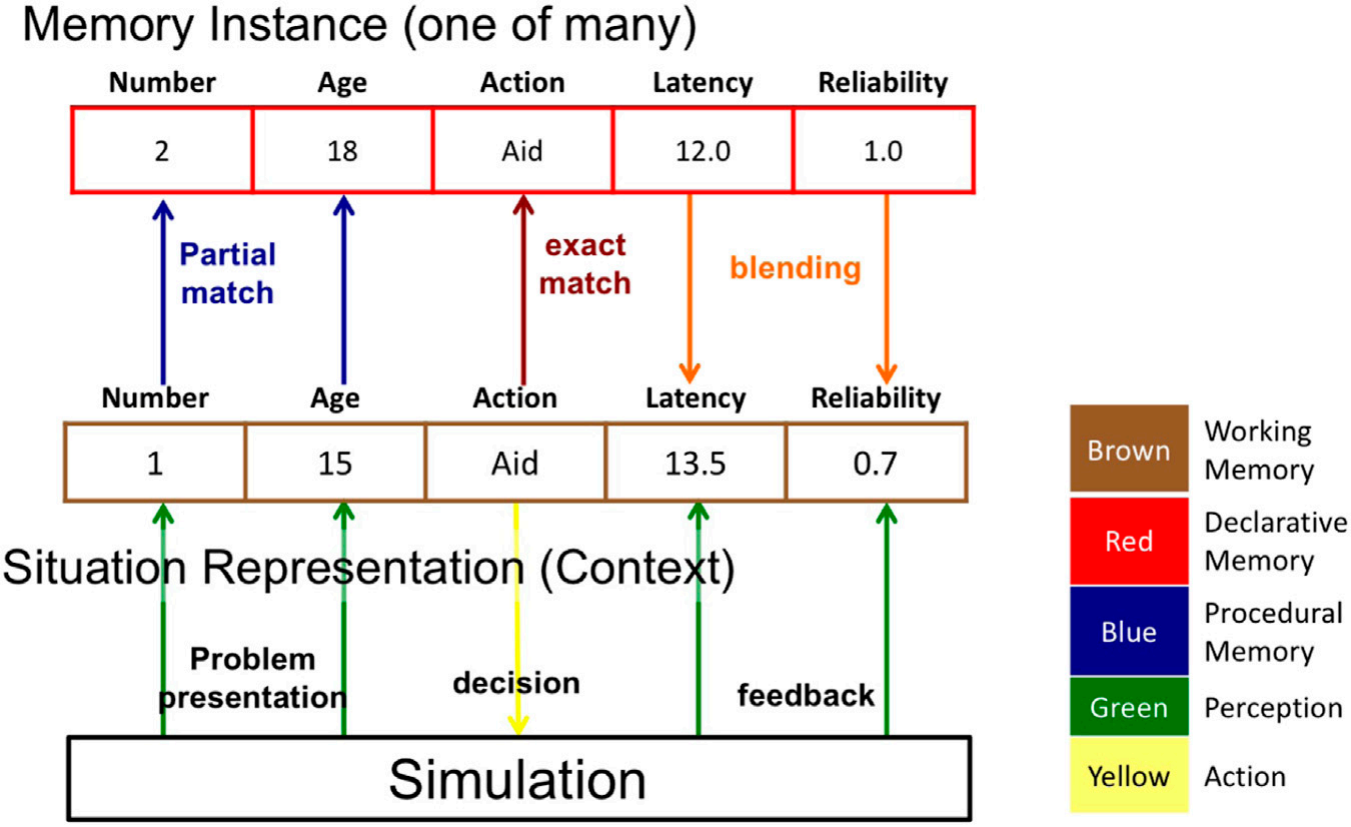
Instance-based learning model representation of the COBALT decisions to rely on automation. The colors used to code each aspect of the IBL model correspond to the type of cognitive module or mechanism leveraged from ACT-R.

Our IBL model adopts a straightforward representation of the COBALT task. Examples are shown in Figure 3, where the middle row is a current trial instance, and the top row is one of many similar instances from declarative memory. The {situation cues} in *i* are the Age and/or Number transparency cues; the decision captured in *i* is the first task phase, the decision whether to rely on the Commander or Automated assist search cue (the Action slot showing *aid* in Figure 3). In this model, the {outcomes} represent two aspects of task performance: whether the Automated assist was reliable (*Reliability* slot) and the total time to complete the visual search (*Latency* slot).

To make a decision about which search assist type to select, the model generates an expected outcome for each assist type by performing blended retrievals for the specific situation feature(s) available (age, number, or both) and each assist type (Automation Aid or Commander), extracting an expected value for total search time. The model then selects the assist type with the lowest expected search time. Note that the activation of each memory instance includes random noise, making the expected value generated by the blended retrieval stochastic, and the decision selected probabilistic. The model then generates an expectation for the reliability of the Automated assist in a similar manner, using a blended retrieval over situation feature(s) and selected assist type. If the automation is expected to be reliable, then it chooses to rely on it, otherwise not. The model then executes the selected option and stores a new instance combining that situation’s feature(s), the option chosen, and the outcomes experienced in terms of reliability and search latency.

We can query the model for its calibration of automation reliability at any time. Usually, we query at the end of a block of trials to give time for experiences to shape the internal representation. At the end of each condition, the model generated a general expectation of reliability through a blending retrieval with no features specified.

### 2.6 Model Initialization

IBL models need either a backup strategy (such as random exploration) to get started or some initial instances to bootstrap the learning process. We chose the latter route, creating three practice instances to represent as broad a range of outcomes as possible. Those instances could have resulted from a short practice phase or fairly straightforward reflection upon the instructions; both instructions and a few practice trials were given to COBALT participants in Fallon et al. (2019). The first initial instance featured the most reliable cues (three sensors and 1-hour-old data), a decision to rely on Automation, outcomes of reliable Automation, and fastest search time (directed search time of 3 s). The second instance featured the least reliable cues (one sensor and 36-hours-old data), a decision to rely on Automation, outcomes of unreliable Automation, and the slowest search time (random search time of 15 s). The third instance featured average cues (two sensors and medium age), a decision to rely on the Commander assist, outcomes of reliability, and an intermediate search time (wait then direct search for a total time of 8 s).

### 2.7 Model Parameters

In our simulation experiments, all parameters were left at their default ACT-R values: decay d=0.5; activation noise s=0.25; mismatch penalty factor MP=1.0; and linear similarities over [0,−1.0].

### 2.8 Reliability Metrics

We measure calibration of reliance with three metrics.

The first metric is a subjective assessment of the Automated assist. For the human participants in Fallon et al. (2019), participants were asked to provide a numeric rate for the reliability of the Automated assist on a scale of 0–100 percent. For the model, this estimate was extracted as the blended value estimate as described above.

The second and third metrics are the positive and negative predictive power statistics, derived from a signal detection theory interpretation of the choice response rates. Positive predictive power is the rate of correctly giving a positive response (true positive rate), out of all the times a positive response was given:

$$\text{PPP}=\frac{\text{True Positive}}{\text{True Positive}+\text{False Positive}}$$ (4)

Negative predictive power is the rate of correctly giving a negative response (true negative) out of all the times a negative response was given:

$$\text{NPP}=\frac{\text{True Negative}}{\text{True Negative}+\text{False Negative}}$$ (5)

We computed predictive power statistics separately for the two task phases because the interpretation of true/false positive/negative is dependent on the nature of the decision being made. In the Decision stage, the decision maker (human or the IBL model) selected between requesting the Automated assist or the Commander assist. Table 1 gives the interpretation of choice types relative to the ground truth reliability of the automation on each trial. From the perspective of calibrating reliance on automation (rather than calibrating reliance on the commander), a true positive is selecting the automation when it was reliable, and so on for the other conditions. Thus, for decisions, PPP provides the degree of calibration of the decision maker to making an Automated assist request when the automation will be reliable, and NPP gives the decision maker’s calibration to selecting the Commander, when the Automation will not be reliable.

**TABLE 1:** Signal detection theory mapping of decision stage.

	Actual reliability of automated assist	
User action	Reliable	Unreliable
Automation selected	True positive	False positive
Commander selected	False negative	True negative

For the Search phase of the task, reliance on automation is defined as following the automation cue in the search task (panning in the direction the cue points). Table 2 gives the signal detection theory response mappings for the Search phase on the trials in which Automated assist was selected in the Decision stage. Note that for this analysis, we only consider the trials where participants had selected the Automated assist in the Decision stage because there was no reliance on automation in the decisions to rely on the Commander assist; a secondary predictive power analysis is possible for reliance on the Commander assist in search, but this was a small subset of trials and does not tell us much about the calibration of automation reliance.

**TABLE 2:** Signal detection theory mapping of search stage.

	Actual reliability of automated assist	
User action	Reliable	Unreliable
Follow automation cue	True positive	False positive
Not follow automation cue	False negative	True negative

Predictive power statistics provide direct metrics of appropriate reliance because they reflect the decision maker’s ability to correctly select the automation when it will be reliable and not select the automation when it will be unreliable, respectively, while accounting for the prevalence of reliable and unreliable trials in the experiment. Accounting for the base rate of reliability is a core part of the definition of trust/reliance calibration. Many studies leverage the metrics $d^{\prime }$ and β (decision criterion) to examine human judgments about the reliability of alarms or automation recommendations (e.g., Bartlett and McCarley, 2019). These metrics emphasize the participants’ abilities to discriminate signal cues from noise or non-signals. Application in the present study would measure participants’ abilities to discriminate the transparency cues indicating the Automation reliability; the emphasis is on how participants internally represent the transparency cues. While this is of interest toward understanding trust calibration, $d^{\prime }$ and β are only indirect indicators of how well someone’s reliance has been calibrated. PPP and NPP give us a direct indicator of calibration. Additionally, there is evidence that PPP and NPP better reflect the time-varying nature of decision-making processes without changing their statistical properties (Repperger et al., 2009).

### 2.9 Blocks and Trials

Each block of task simulation included 143 trials, matching the trial numbers used by Fallon et al. (2019). Twenty simulations per condition were run, for model sample sizes comparable to the total number of human participants in the earlier study (N=16).

## 3 Results

**FIGURE 4:**
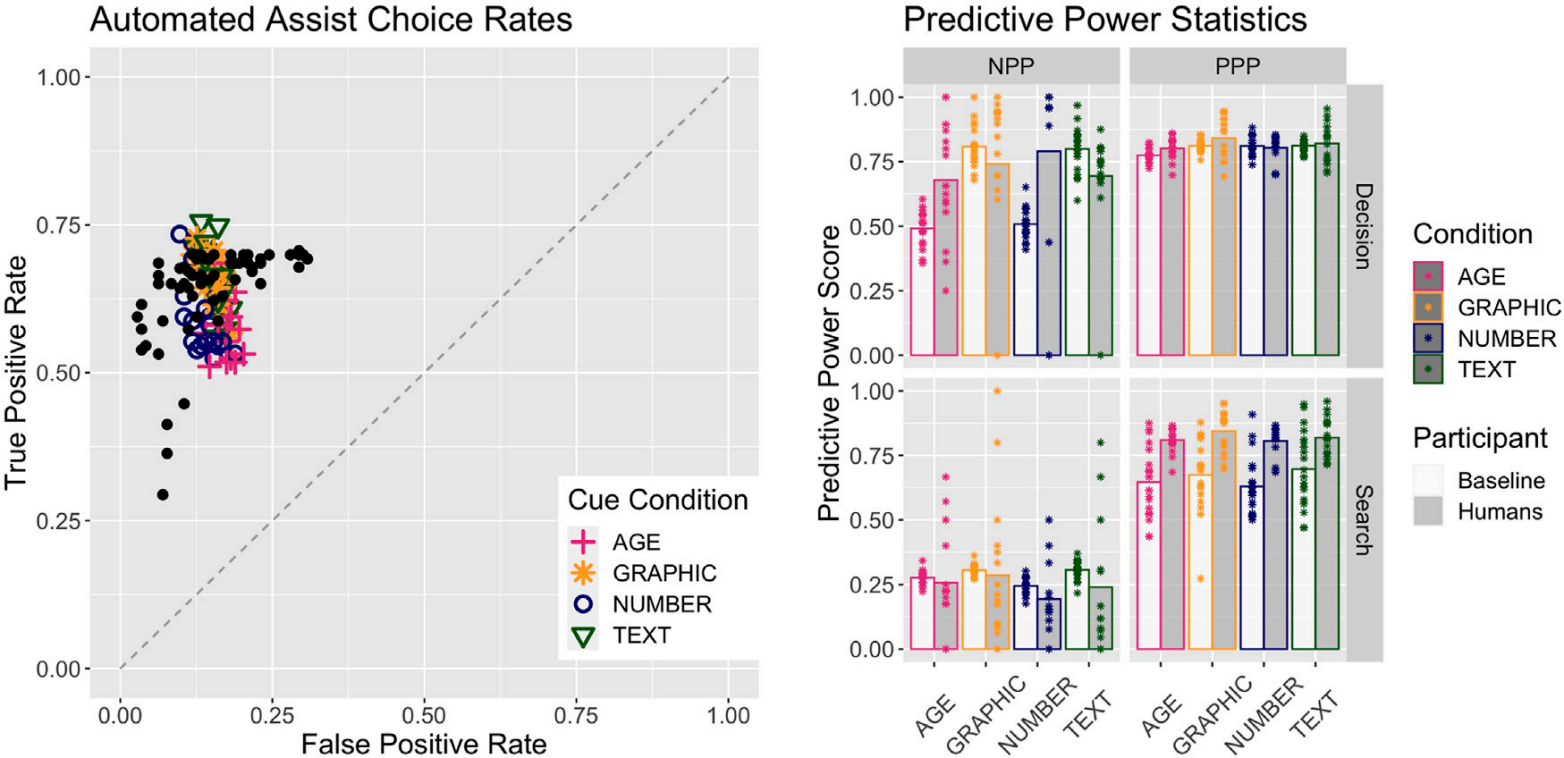
Performance of the Baseline model together with human performance. Human data are from Fallon et al. (2019). Left plot shows the plot of True Positive against False Positive scores; colored icons are the model performance for each condition, and the black dots are the human data overlaid. The right plot gives the predictive power scores for the humans and the Baseline model in both phases and all conditions. Bar heights are the mean values and the points represent either one person or one iteration of the Baseline model.

Figure 4 describes the results of the Baseline version of the model presented earlier. Note that the plot includes the human data from Fallon et al (2019) that was used to develop this model in Blaha et al (2020), plotted as black points. The model selected the Automation about 72% of the time in the Age condition, 67% of the time in the Number condition, and 82% of the time in both the Text and Graphic conditions. This indicates a higher reliance on automation when both transparency cues are presented. The assist type Decision stage decision’s mean PPP value was 0.77 for the Age condition, 0.79 for the Number condition, and 0.82 for the Text and Graphic condition, indicating that reliance on automation was also more accurate when both features are presented. More significantly, the Decision stage decision’s mean NPP value was 0.48 for both Age and Text conditions, but 0.81 for both Text and Graphic conditions. This indicates a much more accurate reliance on the Commander and lack of trust in Automation when both transparency cues are presented.

Patterns are similar but more muted for the Search stage’s search cue reliance decision. The reliance decision’s mean PPP values are 0.60 for the Age condition, 0.62 for the Number condition, and 0.72 for both Text and Graphic conditions. The mean NPP values are 0.28 for Age and Number conditions and 0.30 for both Text and Graphic conditions.

The mean estimates of automation reliability are 0.77 for the Age condition, 0.80 for the Number condition, 0.80 for the Text condition, and 0.81 for the Graphic conditions. These are roughly in line with the respective PPP values, indicating a generally accurate and well-calibrated judgment of automation reliability. Reliability estimates from the model are given in Table 3, which also includes the subjective human ratings from Fallon et al. (2019) for reference.

**TABLE 3:** Reliability ratings.

Condition	Age		Number		Text		Graphic	
	Mean	(SD)	Mean	(SD)	Mean	(SD)	Mean	(SD)
Human ratings	0.75	(0.14)	0.74	(0.12)	0.82	(0.11)	0.80	(0.15)
Baseline	0.77	(0.06)	0.80	(0.16)	0.80	(0.06)	0.81	(0.07)
Frontload	0.82	(0.07)	0.81	(0.09)	0.81	(0.06)	0.83	(0.06)
Frontload-Commander	0.69	(0.11)	0.77	(0.11)	0.73	(0.08)	0.74	(0.06)
Frontload-Positive	0.73	(0.09)	0.80	(0.10)	0.81	(0.06)	0.79	(0.07)
Midload	0.74	(0.08)	0.79	(0.10)	0.78	(0.06)	0.77	(0.07)
Random	0.78	(0.07)	0.82	(0.06)	0.74	(0.13)	0.79	(0.06)

### 3.1 Cue Transfer Impacts

**FIGURE 5:**
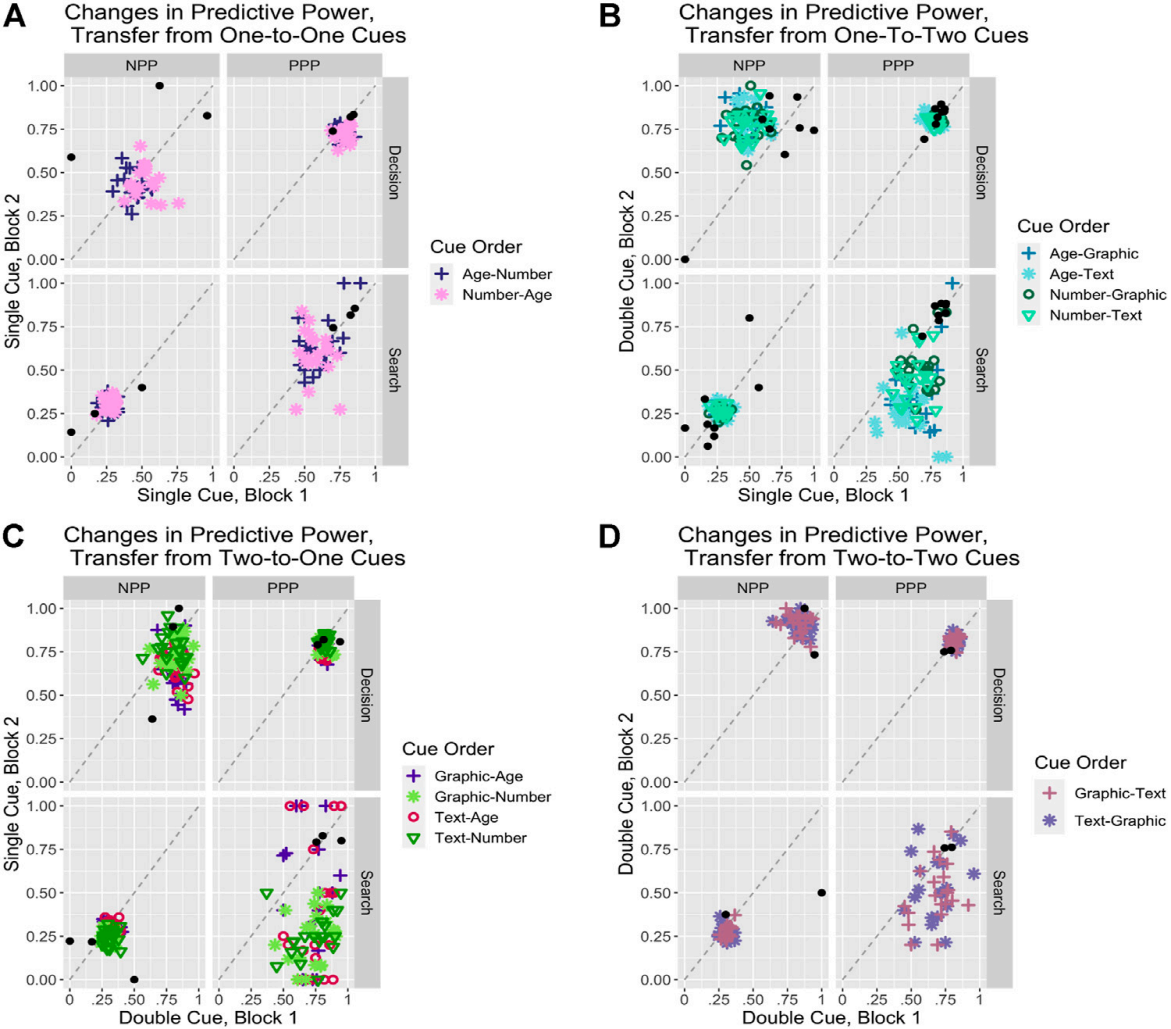
Predictive power statistics plotted for the cue transfer manipulations.

The first set of manipulations concern transfer of trust and reliance across different levels of transparency and information provided to the user. Figure 5B describes the effects of transfer from conditions that present one of the transparency cues (either age of data or number of sensors) to conditions that present both of them (either in the text or graphic form); Figure 5C shows the opposite direction of transfer from two cues to one. The model’s predictions for transfer between conditions reflects the IBL approach of decisions by experience. All experiences are stored in the same memory structures regardless of content. This allows the model to use all instances to make decisions, regardless of which condition they were accumulated under. However, the activation processes determine how those instances are weighted to generate the expectations that lead to decisions using the blending mechanism. As the model transitions from one condition to another, memory instances from both conditions compete on the basis of activation. Instances from the previous condition initially have the advantage of higher frequency and dominate accordingly but will decay over time. Instances from the new condition are initially few but have the advantage of recency and gradually take over as their number increases and instances from the previous condition decay further. Consequently, the transfer between conditions will reflect changing generalization patterns as one set of instances gradually comes to dominate over another.

We will look first at the transfer from single-cue conditions to double-cue conditions (Figure 5B). A basic question is how do the accuracy patterns of the lower information condition transfer to the higher information condition? While the transfer is almost perfect in cases when the automation is reliable, the impact is significant in cases when the automation is unreliable. In those cases, while the choice between Automation and Commander is evenly split in the Baseline data, the transfer in this condition results in choosing the unreliable automation almost 60% of the time. This results in a slight decrease in decision PPP and NPP by a few percent but an especially sharp decrease in search PPP, and to a lesser extent in search NPP, compared to the Baseline. This pattern is more pronounced in the transfer from the Age compared to the Number condition, presumably because Number involves greater accuracy than Age (36 values vs. 3). In summary, increasing automation transparency and the information available improves performance, but the time spent with lower transparency and information does carry a cost.

Conversely, the question when transitioning from the higher information double-cue condition (Figure 5C) is whether the more accurate patterns will improve performance or introduce biases in the lower information condition. The primary effect is to significantly increase reliance on the Automation by about 13–15% from about 72 to 85% for Age and from about 67 to 82% for Number. That increase happens in all conditions but is more pronounced in cases when the automation is reliable. That leaves decision PPP relatively unchanged but significantly improves decision NPP from about 0.47 to 0.65 for Age and from about 0.49 to about 0.74 for Number, reflecting a much more targeted use of the Commander choice. In summary, prior training with a high transparency system retains benefits and improves performance when some of that information is no longer available.

**FIGURE 6:**
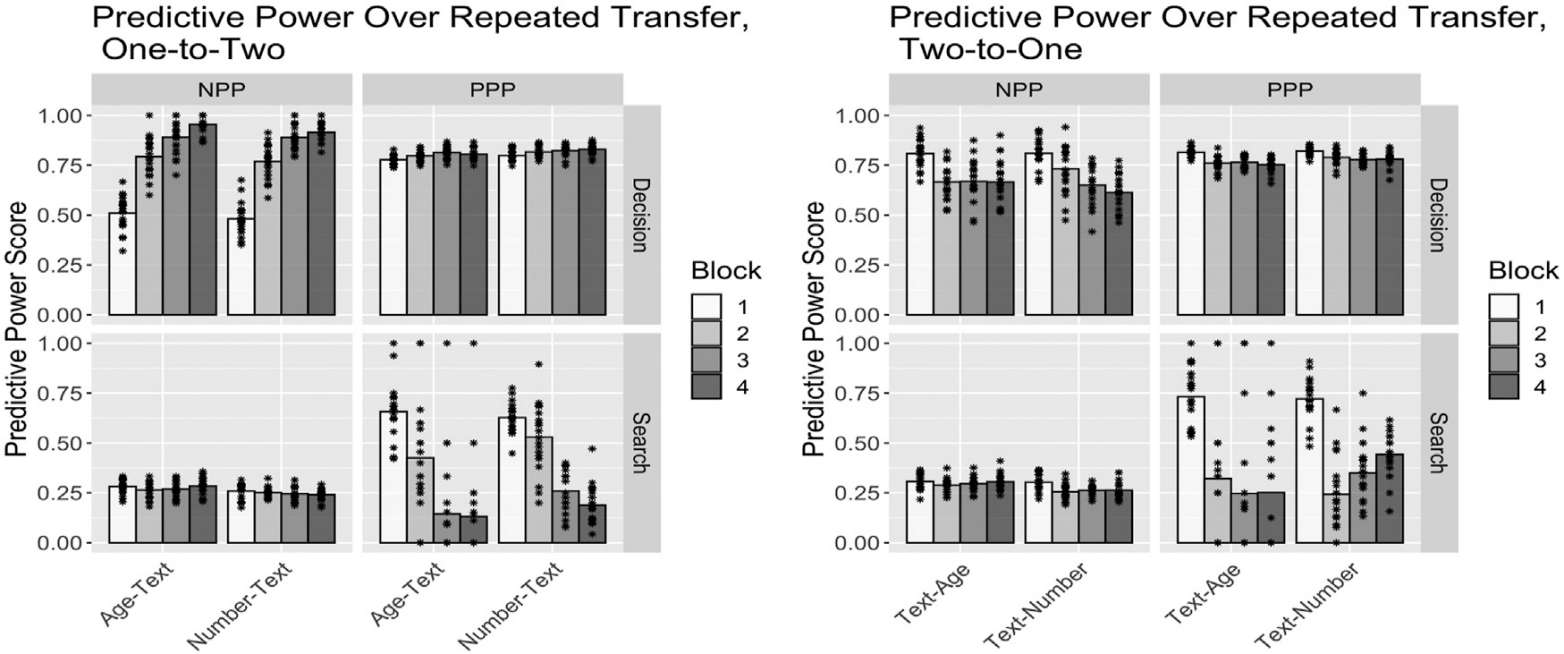
Plots of predictive power statistics over repeated transfer, where the second cue type is repeated for three blocks total. Bars are the means and points are the individual model scores.

We have just seen that transitioning from low to high information carries costs, while the reverse transition from high to low information carries benefits. An important question is whether those transition costs and benefits decay over time or retain some asymptotic value. To determine that, we ran a variant of the transfer manipulations above, where we repeated the second condition for another two blocks, replicating the full length of the original study. Figure 6 summarizes those results. For transfers from single-cue conditions to double-cue conditions (i.e., low to high information/transparency), the main long-term effect is an increased use of the Automation when it is reliable and the Commander when it is not, resulting in both a slight increase in PPP from about 0.81 to about 0.84 and a much more substantial increase in NPP from about 0.81 to about 0.93 over the Baseline. Results for transfer in the opposite direction are more mixed: for both Age and Number conditions, the main effect is an increased use of the Automation across the board, resulting in relatively unchanged decision PPP values but significantly improved decision NPP values by about 20%. In summary, in both cases, we observe significant long-term benefits from training in one condition before transitioning to a different long-term condition providing either more or less information and transparency.

### 3.2 Disrupting Cue–Behavior Relationship Impacts

**FIGURE 7:**
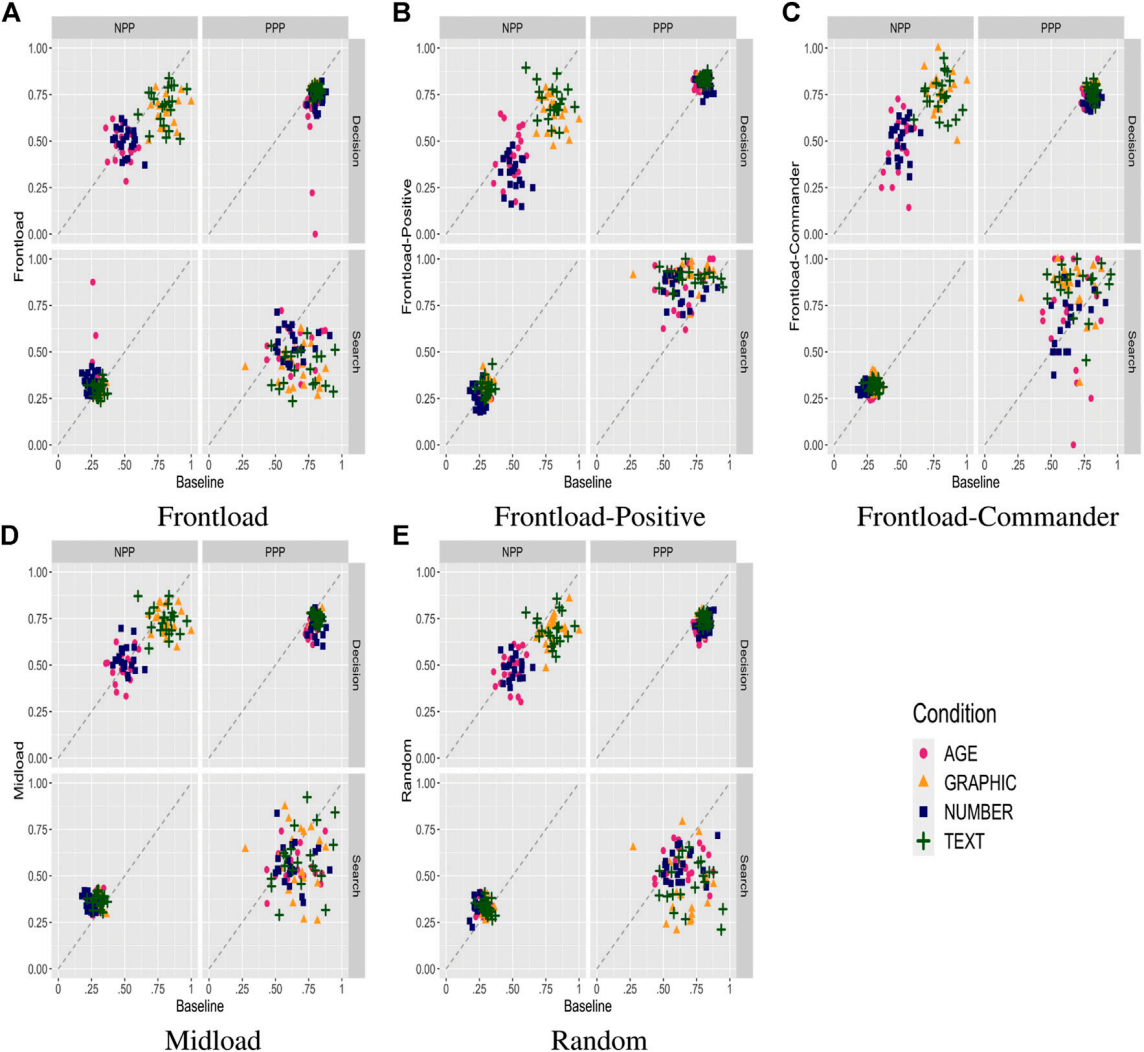
Predictive power statistics plotted with the Baseline condition scores on the *x*-axis and one of the cue-performance disruption manipulations on the *y*-axis. The subplots capture the different manipulations. **(A)** Frontload. **(B)** Frontload-Positive. **(C)** Frontload-Commander. **(D)** Midload. **(E)** Random.

The second set of manipulations concerns the impact on trust calibration of attacks or disruptions of the performance and transparency information. Those attacks will take the form of disruptions (either degrading or upgrading) to the performance of the Automation (or the Commander) at various points in the experiment. Figure 7 summarizes the predictive power in both decision and search phases for the various manipulations. The bulk of the effects are in the Decision stage, and we will focus on that aspect of the data in the following description.

The first manipulation (Random) is to introduce a probabilistic disruption (10% of the time) when the automation is selected and otherwise reliable. The effect is to render the automation unreliable in those cases, which account for about 7% of total trials, decreasing the overall reliability from 70% to about 63%. Figure 7E summarizes the impact on predictive power measures for each of the four transparency conditions. The primary effect is to raise the probability of choosing the Commander option in all conditions by about 12% in the Age condition (from 28 to 40%), 8% in the Number condition (from 32 to 40%), and 8% in the Text and Graphic condition (from 19 to 27%). The increased reliance on the Commander is across the board, and the impact on decision accuracy is mixed, with PPP decreasing by about 0.07 in all conditions and NPP unchanged for the Age and Number conditions and decreasing by about 0.1 for the Text and Graphic conditions. Reliance PPP was slightly lower in the low information conditions (Age and Number) and more substantially impacted in the high information conditions (Text and Graphic), reflecting the comparative impact of random disruptions. In summary, a randomly distributed disruption of automation reliability results in a roughly proportional increase in shift away from automation in all conditions, indicating that the model behaves quite rationally in its calibration of trust in automation.

A fundamental question is whether there are disruptions that can be more strategically targeted to impact trust in automation. One possible manipulation is to mass the disruptions to the automation rather than spreading them throughout the trial blocks. The first option, Frontload, aims to take advantage of primacy effects to disrupt trust before it is fully established by concentrating disruptions is the first third of trials. The disruption probability is tripled to 30% to make it directly comparable to the Random condition in terms of an equal amount of disruptions. Figure 7A summarizes the impact on predictive power measures for each of the four transparency conditions. Even though the automation reliability averaged over the entire period is exactly the same as for the Random condition, use of Automation is significantly impacted, with the probability of choosing the Commander increased 9% in the Age and Number conditions (from 40 to 49%) and 7% in the Text and Graphic conditions (from 27 to 34%) compared to the Random condition. As with the Random condition, the shifts happen across the board, with decision PPP slightly increasing in all conditions and decision NPP decreasing for the Text and Graphic conditions. Estimates of automation reliability also decreases significantly in all conditions.

A third manipulation, Midload, is similar to Frontload but is focused on the middle third rather than the first third of trials. Figure 7D summarizes the impact on predictive power measures for each of the four transparency conditions. Unlike Frontload, results in Midload are substantially similar to Random, confirming that the greater disruption achieved by Frontload that resulted from exploiting a lack of existing trust at the start of the experiment rather than the concentration of disruptions per se.

A fourth manipulation, Frontload-Positive, is similar to Frontload but increases the reliability of automation rather than decreasing by the same amount of 7%, up to 77% from 70%. While such a manipulation would seem counter-intuitive, raising trust in automation above a level that would be supported might be equally disruptive, preventing users from properly relying on alternative options when appropriate. Figure 7B summarizes the impact on predictive power measures for each of the four transparency conditions. Compared to the Baseline, reliance on the Commander decreases by 12%, from 28 to 16%, in the Age condition, by 10%, from 32 to 22% in the Number condition, and by 6%, from 19 to 13%, in the Text and Graphic conditions. However, the shift in reliance away from the Commander happens largely from unreliable conditions, leading to a slight increase in decision PPP but a significant decrease in decision NPP. Estimates of automation reliability are largely unchanged because the artificial increase in reliability is canceled by the induced reliance on automation in conditions where it is not usually warranted. In other words, increasing automation reliance in situations where it is not normally reliable can lull the user in using it more in those conditions, leading to increase in unwarranted use.

A fifth and final manipulation, Frontload-Commander, is similar to Frontload but disrupts the reliability of the Commander instead of the Automation. In its intended effect, it is similar to Frontload-Positive in its attempt to raise unwarranted use of Automation, but even more effective. Figure 7C summarizes the impact on predictive power measures for each of the four transparency conditions. Compared to the Baseline, reliance on the Commander decreases by 18%, from 28 to 10%, in the Age condition, by 17%, from 32 to 15% in the Number condition, and by 9%, from 19 to 10%, in the Text and Graphic conditions. The sizable shift in reliance on Automation across all conditions results in a drop in decision PPP by about 6% for all conditions and a similar 6% decrease in decision NPP for the Text and Graphic conditions only. Estimates of automation reliability drop by about 10% across all conditions. As with Frontload-Positive, but even more effectively, unduly increasing (relative) trust in automation by strategically degrading performance of alternatives can lead to a prolonged overreliance on automation.

## 4 Discussion

We introduce a general methodology for studying effects of trust calibration and reliance in automation. We developed a cognitive model representing the cognitive processes involved in interacting with automation, learning from experience to estimate its trustworthiness, then deciding whether to rely on it or on other sources such as a human expert. Once the model is validated against experimental data, computational experiments can then be run to explore manipulations that can impact or distort trust calibration and reliance on automation. Additional human subject experiments can then be run to confirm the model predictions in the conditions of greatest interest.

Human users develop trust in automation in an incremental way. They follow a process of sequential decision-making in which information gradually accumulates, intermediate judgments are made, and additional information is sought and integrated to yield revised judgments. These iterative sensemaking processes have been modeled using the same dynamic activation processes used to develop computational accounts of cognitive biases such as anchoring and adjustment (Lebiere et al., 2013) and confirmation bias (Cranford E. A. et al., 2020). Once biased judgments lead to decisions such as whether to rely on automation, emergent phenomena such as risk aversion can emerge from self-fulfilling biased sampling processes (Lebiere et al., 2007).

In this work, we posed two exploratory questions to probe the potential for trust calibration to be a source of potential vulnerability or a target for adversarial disruption of the human–machine system. First, we probed how changes in transparency cues, through adding or removing cues, may influence appropriate reliance. Second, we probed how disruptions in the relationship between transparency cues and the actual behavior of the automation can be manipulated. In both types of situations, we found ways to artificially increase and decrease appropriate reliance. We also showed the strength of the impact depended on the amount and nature of the prior experience with the cues, both types of cues and diagnosticity of cues for reliable behavior, that is, we predict that calibration of trust through transparency cues can be manipulated to cause automation overreliance and under-reliance, or automation misuse and disuse, respectively (Parasuraman and Riley, 1997).

We note that a limitation of the current approach using the COBALT interface and automation-aided search task is that the single interface does not currently separate the dimensions of actual automation performance reliability and the user’s perception of the automation’s reliability or performance as captured through the interface (including transparency cues, observed automation failures). If a human user can directly observe the automation failure and so learns about the automation’s actual performance through direct experience, the user may have a different calibration of reliance on the automation from the reliance on the cues or system interface. Direct experience with automation system degradation, separable from the interface, may be more common when a person is directly interacting with a robot, such as in the use of personal assistance robots. Here, the observation of the machine may influence a user’s reliance on it without influencing reliability perception of interface elements. The inverse is also possible, in that perceived reliability of the interface can be undermined when it communicates information different from the direct observations. Direct observation is often hard for unmanned vehicle settings where line of sight to the vehicle may be limited or not possible; our current work that manipulates only interface elements applies here. In such remote automation control settings, humans may have no or only delayed additional information about the system to add to their knowledge about the automation vs. interface system components. Future work may benefit from further exploration of the impacts of asynchronous or time-delayed feedback about automation performance. Teasing out these differences may be important for training or mitigation as we use such systems over time. However, from the perspective of an adversary, undermining appropriate calibration through any of these direct or indirect avenues is a success.

The key for our modeling purposes is that degradation is limited, and our purpose was to explore the most effective way for an attacker to make use of that limited attack capability. From that perspective, the attacker has two potential desirable outcomes: 1) under-reaction where the user keeps relying on the automation while it is degraded and 2) overreaction where the user gives up on the automation entirely even though the degradation is limited. The proper trust calibration would be to stop relying on the automation in circumstances where it is effectively degraded but to continue relying on it when the degradation stops. But as we have seen in the present simulations, the recovery of calibrated reliance is not instantaneous. If an operator were to fail to detect an attack or simply underreact to one, then automation reliance decision rates would not change during or after; we would predict that this would cause even decision rates but a drop in the positive predictive power rates. This would be due to an increasing number of false positives among the steady rate of automation reliance choices. Overreaction would be observed in the negative predictive power rates. In this case, the observer would stop relying on the automation altogether; this increases the number of Commander reliance decisions. After an attack, this would be evident as both a change in the relative rates of Automation/Commander decisions and an increased number of false negatives, decreasing the negative predictive power. These predictions would be testable through future empirical work.

Once computational cognitive models used in simulation, as we have here, have improved our understanding of biases and breakdowns in trust calibration, they can be used to develop automated systems to alleviate those threats to effective human–machine teaming. However, human decision makers have numerous individual differences in knowledge, strategies, and cognitive capacity that automated systems have to be sensitive to. Cognitive models tuned to the specific experience and decisions of individuals have been developed to control personalized automated processes, such as defensive cyber deception, in which the model generates a deceptive signal that balances the potential benefits of deceiving a potential attacker against the costs of rebuilding trust if the signal is exposed as deceptive (Cranford E. et al., 2020). The cognitive model developed in this study could therefore serve as the basis of a system designed to limit the damages of attacks and disruptions and properly calibrate human trust in automation. That system could also be used to optimize the complexity of information presented and find the optimal level of transparency by leveraging techniques such as measures and visualization of cognitive salience developed for explainable AI systems (Somers et al., 2019; Cranford E. et al., 2020). In conclusion, cognitive models provide promising computational tools to understand, manage, and calibrate trust and reliance in automation.

## Data Availability

The raw data supporting the conclusions of this article will be made available by the authors, without undue reservation.
